# Glial cell induced neural differentiation of bone marrow stromal cells

**DOI:** 10.1515/med-2020-0229

**Published:** 2020-09-30

**Authors:** Qiang Ma, Ming Cai, Jing-Wei Shang, Jun Yang, Xin-Yi Gu, Wen-Bo Liu, Qing Yang

**Affiliations:** School of Life Science and Biotechnology, Dalian University of Technology, No. 2 Linggong Road, Dalian 116023, Liaoning Province, China; Department of Neurology, Affiliated Zhongshan Hospital of Dalian University, Dalian 116001, Liaoning Province, China

**Keywords:** Alzheimer’s disease, bone marrow stromal cells, cell therapy, glial cell-derived neurotrophic factor, neuronal differentiation

## Abstract

**Background:**

Bone marrow stromal cells (BMSCs) have an important application prospect in the field of cell therapy for various neurodegenerative diseases, and inducing factors that regulate BMSC differentiation are proposed as a promising therapeutic strategy. In this study, we explored the effect of glial cell-derived neurotrophic factor (GDNF) on the course of BMSC differentiation.

**Methods:**

BMSCs were isolated from rat bone marrow and induced by GDNF. The effects of GDNF on BMSC viability and proliferation were verified by cell counting kit-8, MTT, bromodeoxyuridine, and flow cytometry assays. Neuronal differentiation from BMSCs was detected by quantitative real-time polymerase chain reaction and immunofluorescence via measuring the expression of several neural specific markers.

**Results:**

Compared to untreated BMSCs, GDNF induced the differentiation of BMSCs into neuron-like cells and enhanced the expression levels of neuronal markers including nestin and NCAM. Moreover, the expression of SCF was suppressed by GDNF stimulation.

**Conclusion:**

GDNF could elevate the differentiation of BMSCs into neuron-like cells and could be considered as an effective candidate cell for future neuroscience research.

## 1 Introduction

Alzheimer’s disease (AD), a chronic neurodegenerative disease with increasing neuronal death, synaptic loss, and dementia, is the most frequent cause of dementia, accounting for 60–70% of dementia cases. Although some medicine may temporarily improve symptoms, there is currently no treatment available to cure AD or reverse the progression of the disease [1]. In recent years, stem cell-based approach has been proposed as a promising therapy for the treatment of AD [2]. Numerous studies indicate that bone marrow stromal cells (BMSCs) have the potential to develop cell replacement therapies for various neurodegenerative diseases [3,4,5]. As opposed to embryonic stem cells or neural stem cells, BMSCs present several superior features, such as easy access and proliferation, without ethical and immunological problems [6]. Therefore, the specific neurons from BMSCs might be an optimal donor cell to replace the same type of neurons lost due to disease.

Increasing evidence has indicated that intracerebral or intrathecal injection of BMSCs could strengthen endogenous neuronal proliferation, synaptic connections of damaged neurons, and functional recovery [3,7,8]. Meanwhile, BMSCs could release or stimulate secretion of trophic factors that significantly contribute to endogenous repairs, via stimulating angiogenesis, reducing oxidative stress, and decreasing apoptosis [9]. Glial cell-derived neurotrophic factor (GDNF) has a potent neuroprotective effect on a variety of neuronal damage and can activate cell signaling pathways that regulate neuronal survival, differentiation, growth, and regeneration [10,11]. Yang et al. [12] indicated that BMSCs overexpressing GDNF offer further effective neuroprotection for rats with intracerebral hemorrhage and neurons exposed to hypoxia/reoxygenation. Moreover, transplantation of BMSCs modified by GDNF showed a greater therapeutic effect than transplantation of native BMSCs [13]. Based on the aforementioned reports, we deemed that GDNF has an important function in the behavior of BMSCs. However, the specific effect of GDNF on the neural differentiation of BMSCs has not been exposed thoroughly. This study revealed the role of GDNF in the growth and differentiation of BMSCs *in vitro*.

## 2 Materials and methods

### 2.1 Animals and BMSC culture

Animal experiments were carried out by following the guidelines of the Animal Ethics Committee of Dalian University of Technology, China. Primary BMSCs, which were isolated from an adult Sprague-Dawley rat, were acquired from the Laboratory Animal Center of the Academy of Military Medical Sciences (Beijing, China). Briefly, the rats were sacrificed by cervical dislocation, and the femora and tibiae were dissected under sterile condition. Then the metaphyses were removed, the bone marrow cavity was exposed, and the bone marrow cavity was irrigated with Dulbecco’s modified Eagle’s medium (DMEM), which includes 10% fetal calf serum, 100 U/mL of penicillin, and 100 U/mL of streptomycin. The cells were gathered and seeded into a 24-well plate at a density of 9 × 10^5^ cells/mL at 37°C with 5% CO_2_. Replace the culture medium every 3 days and discard the non-adherent cells.

### 2.2 GDNF treatment

BMSCs were plated onto a six-well plate (Corning Inc, Acton, MA, USA) with an initial seeding density of 1 × 10^4^ cells/cm^2^ in regular growth medium for expansion purpose. For differentiation study, GDNF (10 ng/mL) was added to the culture medium, and no GDNF was considered as the control group. The supplemented medium was changed once every 3 days. Cell activity and morphological characteristics were detected under an inverted microscope. Six replicate wells were used for subsequent analysis after 1, 7, 14, and 21 days.

### 2.3 Cell viability assay

Cell counting kit-8 (CCK-8; Bestbio, China) reagent was added to evaluate cell viability. Briefly, the cells were implanted in a 24-well plate at a density of 2 × 10^4^ cells/well. The culture medium was removed, and the cells were washed with phosphate-buffered saline (PBS) three times. Then, CCK-8 reagent was added into each well, followed by incubation for 2 h under the aforementioned condition. The supernatant was transferred to a 96-well plate, and the optical density (OD) was read with a microplate reader at the wavelength of 450 nm.

### 2.4 Cell proliferation assay

Cell proliferation analysis was performed by two ways, bromodeoxyuridine (BrdU) analysis for changes in cellular DNA synthesis and MTT assay for changes in cell numbers. The MTT assay was executed as described by Mosmann [14]. First, the cells were plated in a 96-well plate. After the cells were washed twice with PBS, BMSCs were exposed to 0.5 mg/mL MTT stock solution (Sigma) at 37°C for 4 h. Then, the MTT solution was removed and replaced with 10% dimethyl sulfoxide to solubilize the formazan crystals at 37°C for 6 min. BrdU is a thymidine analog that is able to be incorporated into DNA of dividing cells during the S-phase. During the period of labeling, BrdU was incorporated into the DNA of proliferating cells instead of thymidine. The BrdU cell proliferation assay was performed by Abcam kit (Abcam, Cambridge, UK) according to the manufacturer’s instructions. In brief, 20 µL of BrdU-labeling solution was added to each well and incubated for 4 h. Afterward, the cells were washed twice with DMEM to remove non-incorporated BrdU, then the cells were fixed with 4% paraformaldehyde and denatured with HCl for 15 min at 37°C. Nucleases were added to the cells to improve the accessibility of the incorporated BrdU for detection by anti-BrdU antibody at 37°C for 30 min. After washing with DMEM three times, the cells were incubated with 5% bovine serum albumin in PBS, followed by incubation with primary mouse monoclonal anti-BrdU (1:200; Abcam) overnight at 4°C. After washing away unbound anti-BrdU using DMEM, samples were treated with FITC-conjugated goat anti-mouse IgG secondary antibodies (1:200, ZSGB-BIO) for 2 h in the dark at room temperature. The cultures were then washed three times for 5 min using PBS. Finally, the cultures were covered with 50% glycerinum. The optical densities of active cells were determined using an Olympus fluorescence microscope (Olympus, Tokyo, Japan) at 495 nm.

### 2.5 Flow cytometry detection

Flow cytometry was used to quantify cell cycle distribution by determining DNA content of cells by propidium iodide (PI) staining. BMSCs were cultured in fresh medium to 90% confluency, harvested by trypsinization, and washed twice with PBS. Then, a volume of 2 mL of cold ethanol was added for immobilization for 24–48 h at 4°C. Next, the cells were washed with PBS, and 0.1 mg RNase A was added for RNA degradation. The cells were then placed in the dark for 30 min after adding 0.2 µg PI. The percentages of cells at different phases of cell cycle were examined by flow cytometry (Becton Dickinson, Heidelberg, Germany).

### 2.6 Quantitative real-time polymerase chain reaction (qPCR)

Total cellular RNA was isolated from the cells in each group using Trizol (Invitrogen, Carlsbad, CA, USA). Then, 1 µg total RNA was reverse-transcribed into the first-strand cDNA utilizing Superscript III reverse transcriptase (Invitrogen, Carlsbad, CA, USA) according to supplier’s specification. Synthesized first-strand cDNA was used as a template, and β-actin was applied as a normalization for PCR amplification, respectively. For qRT-PCR analyses, amplification reactions were implemented by means of ABI Prism 7500 sequence detection system (Applied Biosystems, Foster City, CA, USA) with SYBR green qPCR ThunderBird (Toyobo, Osaka, Japan). The specific primer sequences were exhibited as follows:

*NCAM* F: 5′-TATCCACCTCAAGGTCTTCGC-3′;

*NCAM* R: 5′-TGTCTTCACTGCTGATGTTCG-3′.

*SCF* F: 5′-CAATAGGAAAGCCGCAAAGTC-3′;

*SCF* R: 5′-GCAGCAAAGCCAATTACAAGC-3′.

*NESTIN* F: 5′-GACCTCCTTAGCCACAACCCTC-3′;

*NESTIN* R: 5′-GATTTGCCCCTCATCTTCCTG-3′.

β-actin F: 5′-CAGGGAAATCGTGCGTGAC-3′;

β-actin R: 5′-GACATTGCCGATAGTGATGACCT-3′.

### 2.7 Immunofluorescence staining

Cultures were fixed with 4% paraformaldehyde in PBS for 15 min at about 25°C, and then the cells were sealed with 5% bovine serum albumin in PBS for 30 min, followed by incubation with primary antibodies, including nestin (1:200; Sigma-Aldrich, St. Louis, MO, USA), NCAM (1:500; Sigma-Aldrich), and SCF (1:200; Abcam), overnight at 4°C. After washing with PBS, cultures were incubated with FITC-conjugated goat anti-mouse (1:200; ZSGBBIO) at about 25°C in the dark for 2 h. Fluorescence signals were detected with an Olympus fluorescence microscope (Olympus).

### 2.8 Statistical analysis

All data were presented as mean ± standard error of the mean and were processed using SPSS 14.0 (SPSS Inc., Armonk, NY, USA). Differences among multiple groups were assessed using analysis of variance (ANOVA) by following the Bonferroni post hoc test. *P* < 0.05 was considered as statistically significant.

## 3 Results

### 3.1 Impacts of GDNF on the viability and proliferation of BMSCs

**Figure 1:**
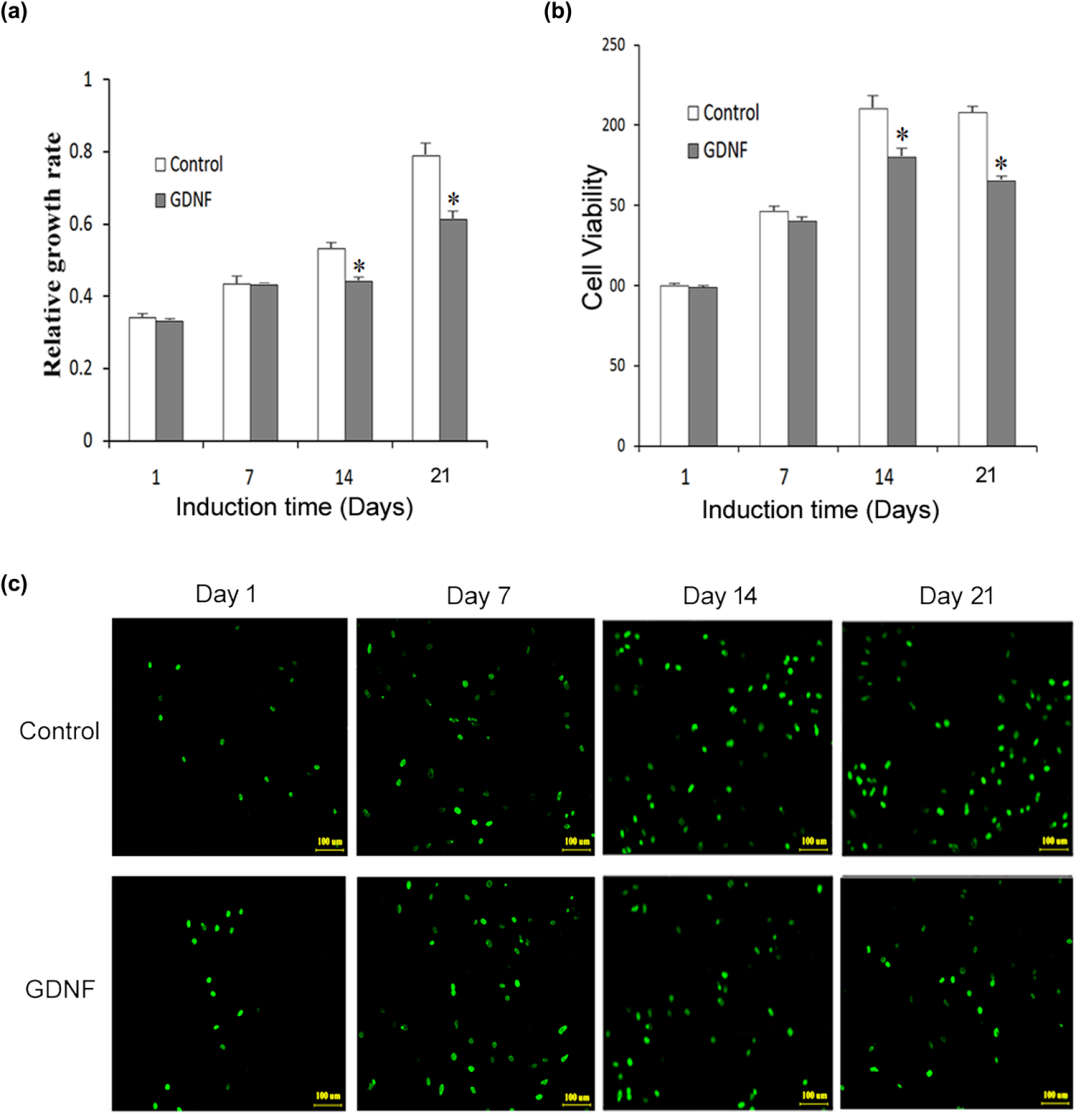
Effect of GDNF on BMSC viability and proliferation. (a) The OD values of bone marrow stromal stem cells were measured by CCK-8 after GDNF induction. (b) Effect of GDNF induction on cell viability of bone marrow stromal stem cells was determined by the MTT assay. (c) FITC-conjugated Brdu (green) immunofluorescent staining. Bar = 100 µm. *p < 0.05 vs control group.

The CCK-8 and MTT assays were applied to measure BMSC activity at different time periods. As shown in Figure 1a (ANOVA: *F*(3, 8) = 87.719, *p* < 0.05), cell viabilities in both the control group and GDNF induction group elevated over initial days. The activity of BMSCs showed no significant difference from days 1 to 7. On days 14 and 21 of incubation, BMSCs induced by GDNF showed lower cell viability than the control group. To further determine the functional role of GDNF in cell viability, the MTT assay was also employed to determine the viability of BMSCs. As shown in Figure 1b (ANOVA: *F*(3, 8) = 159.245, *p* < 0.05), viable cells increased over time in both groups. There was no significant difference of viable BMSCs between two groups from days 1 to 7. After incubation at days 14 and 21, BMSCs in the GDNF group showed lower cell viability than the control group.

Based on the results of viability measurement, in order to better confirm the biological role of GDNF in BMSC proliferation, we utilized the BrdU assay to evaluate the cell proliferative capability through detecting DNA synthesis of BMSCs after GDNF treatment. As presented in Figure 1c, at the beginning of induction, the amount of BrdU incorporation was a common level in both two groups. In the control group, BrdU levels increased over time, relative to that on day 1. In the GDNF induction group, the amount of BrdU-positive cells increased on days 7 and 14 and reduced on day 21. BrdU-positive cells in the GDNF group were significantly less than those in the control group on day 21.

The above experimental results proved that GDNF could decrease the proliferation of BMSCs on day 14 or day 21 of induction time, which may be related to the enhanced differentiation capacity of GDNF.

### 3.2 Effect of GDNF on cell cycle of BMSCs

**Figure 2:**
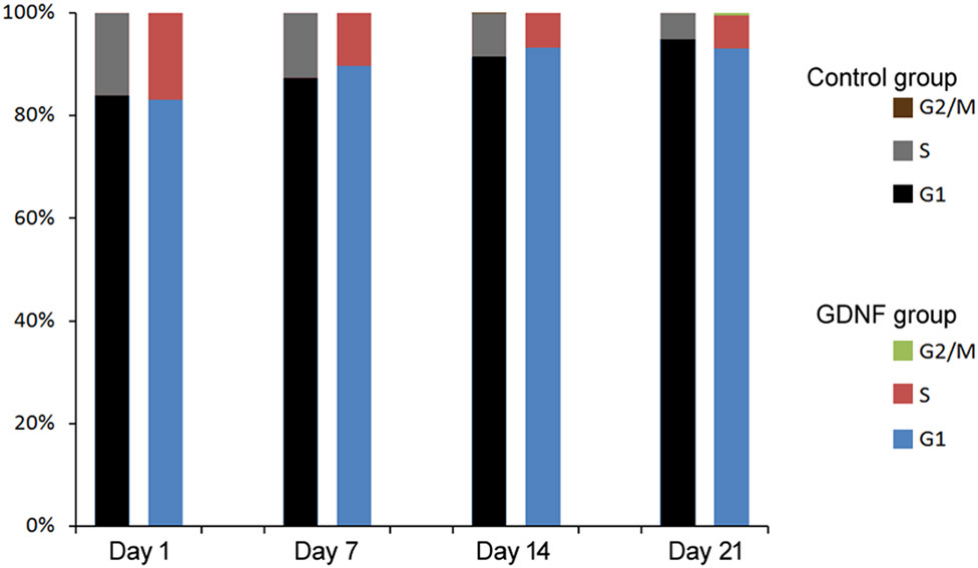
Effect of GDNF on BMSC cell cycle. Cell cycle analyses of bone marrow stromal stem cells were performed by flow cytometry. *p < 0.05 vs control group.

In an attempt to assess the effect of GDNF on BMSC cell cycle, flow cytometry was applied to quantify cell cycle distribution. As shown in Figure 2, with the passage of time, the S phase of BMSCs decreased and the G1 phase increased in both groups. Compared with the control group, the GDNF group had more G1 phase cells and fewer S phase cells, but the difference between two groups was not statistically significant. These data indicated that GDNF could not promote the proliferation of BMSCs.

### 3.3 Induction effect of GDNF on neuron differentiation from BMSCs

**Figure 3:**
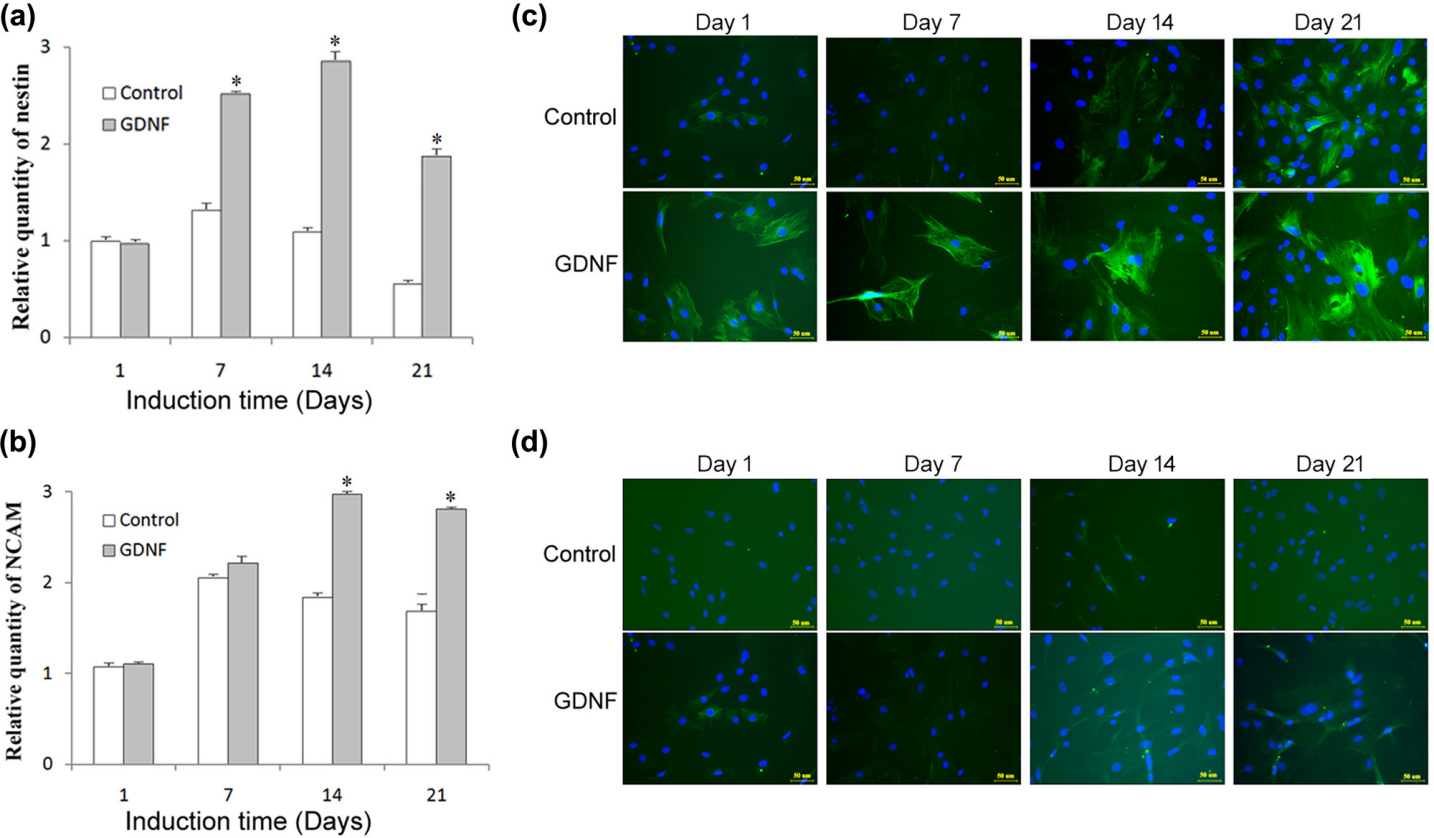
Detection of the levels of nestin and NCAM. (a and b) Expression of nestin and NCAM under GDNF induction were examined by qRT-PCR. (c and d) Expression of nestin and NCAM also analyzed by immunofluorescence. *p < 0.05 compared with the control group.

To discern the cell population rendered from BMSC differentiation caused by GDNF, the expression of several neuronal biomarkers, such as nestin and NCAM, were detected by qRT-PCR and immunofluorescence analysis in GDNF-treated BMSCs. Nestin and NCAM are the early neuronal-associated markers, which are highly expressed in neuronal precursor cells and decreased along with neuronal maturation. As illustrated in Figure 3a (ANOVA: *F*(3, 8) = 3.451, *p* < 0.05), qRT-PCR showed increased mRNA expression of nestin on days 7, 14, and 21 in the GDNF group, and the mRNA expression of nestin on day 21 was lower than that on day 14. Compared to the control group, BMSCs with GDNF treatment demonstrated significantly increased mRNA expression of nestin on days 7, 14, and 21 after stimulation. As shown in Figure 3b (ANOVA: *F*(3, 8) = 18.912, *p* < 0.05), qRT-PCR showed significantly increased mRNA expression of NCAM on days 14 and 21 in the GDNF group (*p* < 0.05), and the mRNA expression of NCAM on day 21 was slightly lower than that on day 14. On day 1, BMSCs showed low expression levels of nestin and NCAM, with no significant difference between two groups.

Simultaneously, immunofluorescence staining images showed similar results to qRT-PCR. As shown in Figure 3c and d, some cells stained positive against nestin and NCAM, and the nestin- and NCAM-labeled cells increased on days 14 and 21 in the GDNF group. Moreover, the morphology of some BMSCs with GDNF induction showed neuron-like changes with multipolar and rounded cell bodies.

### 3.4 Functional role of GDNF in SCF

**Figure 4:**
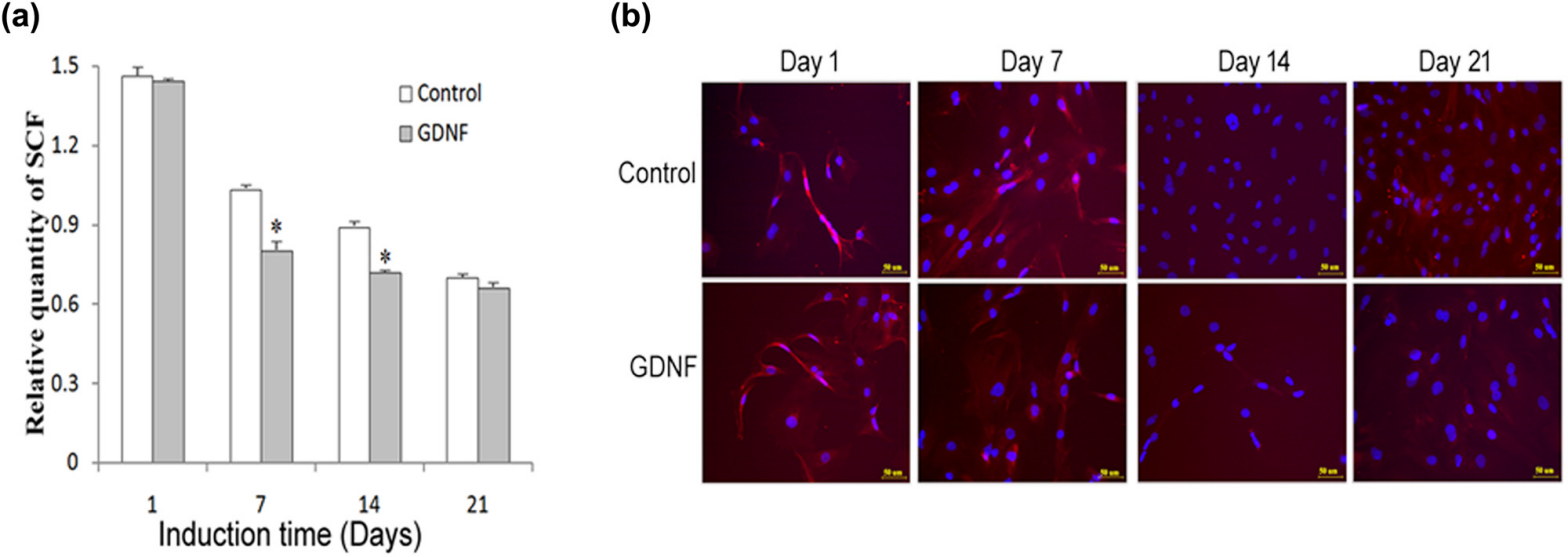
Detection of the level of SCF. (a) The gene expression of SCF in bone marrow stromal stem cells with GDNF treatment was estimated by qRT-PCR. (b) Immunofluorescence analysis of GDNF-treated bone marrow stromal stem cells. GDNF treatment decreases the number of SCF-positive cells. *p < 0.05 compared with the control group.

SCF is identified as an important hematopoietic growth factor for the growth and proliferation of BMSCs. The level of SCF shows a positive correlation with the viability of BMSCs. Thus, we detected the expression of SCF here. QRT-PCR and immunofluorescence staining showed that GDNF treatment resulted in decreased mRNA expression of SCF and decreased SCF-labeled cells (Figure 4a [ANOVA: *F*(3, 8) = 22.805, *p* < 0.05] and Figure 4b).

## 4 Discussion

Regeneration of human nervous system from neurodegenerative disease is a challenge for stem cell-based therapeutic paradigms [15]. BMSCs have been assessed as potential candidates for cell therapy, and many studies have reported that BMSCs can be induced to differentiate into neuronal cells *in vitro* using a variety of protocols [16,17,18]. Despite the advance in BMSCs, no standard protocol has been proposed for the differentiation of neuron-like cells from BMSCs. Previously, chemical reagents were reported to induce BMSC differentiation into neuron-like cells with high productivity [19]. However, the defects of chemical treatment, such as chemical cytotoxicity, high cell mortality, and spurious neuronal differentiation, hampered the clinical approval of chemical-induced cell differentiation [20]. Therefore, it is particularly important to investigate the safe and effective stimulus factors. Currently, neurotrophic factors represent a promising alternative to induction protocols, which can stimulate cell signaling pathways, thereby modulating the survival and differentiation of neurons [21]. In the present study, we revealed that GDNF could promote the growth of BMSCs and preliminarily induce the differentiation of BMSCs into neuron-like cells. These resultant neuron-like cells can form neurosphere-like structures and express early neural cell markers.

GDNF is a potent neurotrophic factor that enhances the survival and morphological differentiation of dopaminergic neurons and prevents the apoptosis of motor neurons [22,23]. These properties make GDNF have potential in the treatment of Parkinson’s disease. In addition, GDNF has been reported to associate with a variety of neural diseases, such as Hirschsprung disease [24], depressive disorder [25], and Tourette syndrome [26]. However, GDNF being a macromolecule could not pass via the blood–brain barrier. Direct infusion of GDNF into the lesion location will cause certain hazard in some circumstances and it is hard to maintain long-term therapy [27]. Subsequently, virus vector-mediated GDNF gene delivery has been developed to obtain sustained delivery [28,29]. Several evidence indicated that persistent overexpression of GDNF might lead to adverse side effects, such as abnormal germination outside the striatum and downregulation of tyrosine hydroxylase in the intact striatum [30]. Therefore, it is crucial to investigate a more effective and safer method for GDNF to function. Importantly, in this study, we discovered that with GDNF treatment, the growth and differentiation abilities of BMSCs were increased, indicating that GDNF may function by regulating the behaviors of BMSCs. Thus, this study found that GDNF has a more long-lasting effect on BMSCs with fewer side effects.

In this study, nestin and NCAM were utilized to verify the neural differentiation of BMSCs. According to the relationship between nestin expression and cell type, nestin is considered to be a common marker of neuronal progenitors in different contexts [31]. NCAM, also known as neural cell adhesion molecule or CD56, is a membrane-bound protein that mediates cell interactions in neurons and is abundant in neuronal precursors [32]. Both nestin and NCAM are expressed mostly in neuronal precursor stem cells, and the expression reduction is consistent with neuronal maturation. Thus, the combination has been extensively used as a marker for neuronal progenitor cells. Significantly, in this study, we observed that the expression of nestin and NCAM showed a significant upward trend from 0 to 14 days and decreased from days 14 to 21, indicating that BMSCs were mature.

Despite we discovered that GDNF promoted the differentiation of BMSCs into neuron-like cells, several limitations of this study should be pointed out. First, the effect of GDNF on BMSCs should be explored. Second, the effect of GDNF on BMSCs should be verified *in vivo*.

Taken together, data on the elements that govern the proliferation and differentiation of BMSCs are accumulating rapidly. In the present research, we indicated the effects of GDNF on the differentiation of BMSCs into neuron-like cells, which may contribute to the application of BMSCs as a useful tool for basic neuroscience study. Despite the advance, it will take time to establish a therapy for AD in clinical study.
